# Study on the energy characteristics of rocks under cyclic loading and unloading

**DOI:** 10.1038/s41598-025-97191-0

**Published:** 2025-04-10

**Authors:** Shaoqing Niu, Jinwen Wu, Jinchang Zhao

**Affiliations:** 1 College of Mining Engineering, Taiyuan University of Technology, Taiyuan, 030024 China; 2 School of Aerospace Engineering, North University of China, Taiyuan, 030051 China

**Keywords:** Dissipation energy, Cyclic loading–unloading, Failure mechanism, Solid Earth sciences, Civil engineering, Mechanical properties

## Abstract

The deep-buried rock masses influenced by prolonged geological processes have accumulated vast amounts of energy. This stored energy is released due to disturbances in the external environment, a series of geological disasters is triggered. In this study, triaxial cyclic loading tests were carried out on sandstone and granite at different strain amplitudes to investigate the variation of rock internal energy. Stress–strain curves were recorded under various amplitudes. The calculation model of dissipated energy, elastic energy and plastic energy was established. Energy variations are analyzed to explore the dissipation mechanism of internal energy in rocks. The results show that with the increase of strain ratio, the dissipated energy, elastic energy and plastic energy all increase. When the strain ratio reaches a certain range, the slope of energy increase exhibits a sudden change. The study of the rock failure mechanism from the perspective of energy offers practical and guiding significance for the safety assessment and stability prediction of various geological disasters.

## Introduction

Geological disasters such as rock bursts, roof instabilities, and collapses occur frequency with increasing underground excavation depth^1–5^. Rocks deeply buried underground remain in a state of equilibrium over long-term geological tectonic activity^6–10^. A substantial amount of energy is stored within them. External engineering disturbances disrupt this balance. Energy release within the rock triggers various geological disasters^11–17^. To address these challenges and analyze the mechanisms of rock deformation and failure, many scholars have conducted research from an energy perspective^18–30^. According to the laws of thermodynamics, the energy transformations within a system reflect its physical state, establishing a connection between system instability and energy changes^31–35^. The energy produced by tectonic activity undergoes conversion into various forms under intricate stress conditions, including elastic energy, plastic energy, dissipative energy, and thermal energy within the rock mass^36,37^.

Examining rock failure from an energy perspective unveils the underlying physical mechanisms of destruction. This approach helps in understanding the root causes of disaster occurrences. Research by Zheng et al.^38^ demonstrated that the presence of strain-softening reduces the rock’s capacity to store elastic strain energy. The energy dissipated and released by the surrounding rock increases exponentially. Tuo et al.’s^39^ experimental results confirmed that after reaching the yield load, energy in coal is dissipated in forms such as plastic deformation, internal damage, block friction, radiation energy, and kinetic energy. Zhang et al.^40^ discovered that elastic strain energy and dissipated energy decrease as the secondary fracture angle increases when the fracture angles are 45° and 90°. Hu et al.^41^ conducted uniaxial compression tests on coal samples at different loading rates. The rockburst energy index K-E was used to calculate the rockburst tendency. They found that the rockburst energy index initially increases and then decreased with loading rate. Li et al.^42^ studied the energy evolution characteristics of rockburst and the formation mechanism of excess energy in rockburst. The study found that the occurrence of rockburst is mainly due to the generation of excess energy, and the excess energy depends on the elastic strain energy stored before rock failure, the external energy input after rock failure, and the residual elastic strain energy. Wang et al.^43^ conducted a series of uniaxial cyclic loading–unloading tests on fine sandstone to investigate the energy evolution under cyclic loading and unloading conditions. The results indicated that the input energy, elastic energy and dissipated energy density exhibited nonlinear growth during the cyclic loading tests. With increasing cyclic loading and unloading duration, the dissipation energy ratio first decreased and then increased. Luo et al.^44^ proposed a calculation method for peak energy density, studied the energy evolution of rock before the post-peak stage under uniaxial compression conditions, and revealed the damage evolution process of rock from an energy perspective. Bai et al.^45^ discovered that the elastic energy accumulated in rock prior to failure decreases with increasing water content. Meng et al.^46^ performed uniaxial cyclic loading and unloading tests on sandstone specimens under six different loading rates. It was revealed that energy accumulation dominates before the axial load reaches its peak strength. Energy dissipation takes precedence beyond this point, with input energy driving the irreversible initiation and propagation of microcracks in the rock. Elastic energy release triggers sudden instability and drives rock damage.

Energy dissipation is a direct manifestation of the initiation, expansion and penetration of microcracks inside rocks. A large number of scholars have conducted cyclic loading and unloading tests to study the energy dissipation mechanism of rocks. Wang^47^ investigated the energy evolution of rocks under graded cyclic loading and unloading, as well as variable lower-limit cyclic loading and unloading paths. The results indicated that the elastic energy, dissipated energy and total energy of the rock were positively correlated with the number of cycles. Under the variable lower-limit cyclic loading and unloading path, the rock experienced more severe damage. Hao^48^ investigated the influence of rock saturation on energy mechanisms during loading and unloading. The results indicated that rock saturation accelerated the increase in dissipated strain energy while mitigating the decline in elastic strain energy. Lu^49^ studied the energy evolution characteristics of rock under different amplitudes and loading–unloading cycles. The results indicated that both the input energy density and dissipated energy density displayed an L-shaped trend, while the elastic energy density showed little variation. Liu’s^50^ research revealed a linear relationship between the input strain energy density and the dissipated strain energy density during the loading–unloading process in rocks. Zhang^51^ et al. employed the area integration method and the superposition method to calculate the elastic energy density, dissipated energy density, and input energy density of rocks under cyclic loading and unloading. These studies reveal the key laws of energy dissipation mechanisms in rocks during cyclic loading and unloading. However, current research on the energy evolution within rocks remains limited. Most studies primarily focus on elastic energy and dissipated energy, the role of plastic energy is neglected. Plastic deformation is a significant component of rock deformation^52^. Plastic energy is incorporated into such studies holds substantial practical value for engineering applications.

The main objective of this study is to investigate the energy dissipation mechanism of sandstone and granite. Three energy calculation models were established based on the stress–strain curves of the rocks. Triaxial cyclic loading tests with variable and constant amplitudes were conducted on sandstone and granite. Energy variation curves were recorded under different strain ratios, confining pressures, and amplitudes. The variation patterns of three types of energy with strain ratio, confining pressure, and amplitude are revealed. This research provides valuable insights into the analysis of energy changes in rocks during geological disasters.

## Test steps

### Rock information

Sandstone is a quintessential sedimentary rock. It formed from materials produced by weathering and erosion of the Earth’s surface which are then transported and deposited. Sandstone primarily consists of sand-sized particles bound together by a cementing substance. Granite is the most common type of igneous rock. It formed through the crystallization of minerals that are chemically bonded together. These two rocks are highly representative and possess distinct structural and compositional characteristics. In this experiment, two types of rocks were selected as research objects to study their structure and composition, thereby providing a deeper understanding of the damping mechanism.

**Fig. 1:**
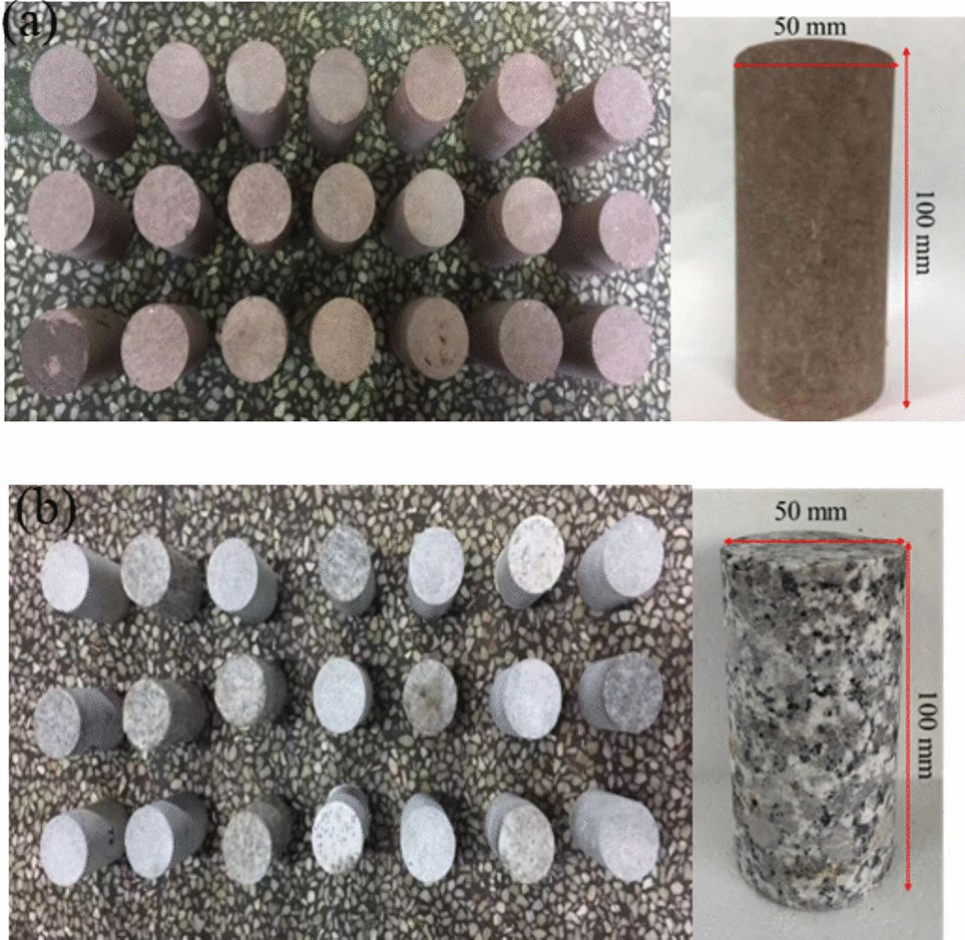
Rock samples (**a**) Sandstone (**b**) Granite.

The specimens selected for this experiment are Permian sandstone and Cretaceous granite. Rock samples obtained from the same geological formation were used to ensure the uniformity of the specimens. After core drilling, cutting and polishing, cylindrical standard samples with dimensions of *ϕ*50 × 100 mm were prepared. The sample has a height-to-diameter ratio of 1:2. The parallelism error of the specimen’s end faces is less than 0.005 mm and the surface flatness error is below 0.02 mm. The side surfaces of the specimens are smooth with a perpendicular diameter error of less than 0.3 mm. Figure 1 depict the sandstone and granite specimens.

They were placed in the same location to air-dry naturally to ensure that the specimens remain in the same condition during the experiment. This stabilizes the moisture content of the sample at a natural level, ensuring consistency during testing.

### Test plans and equipment

**Fig. 2:**
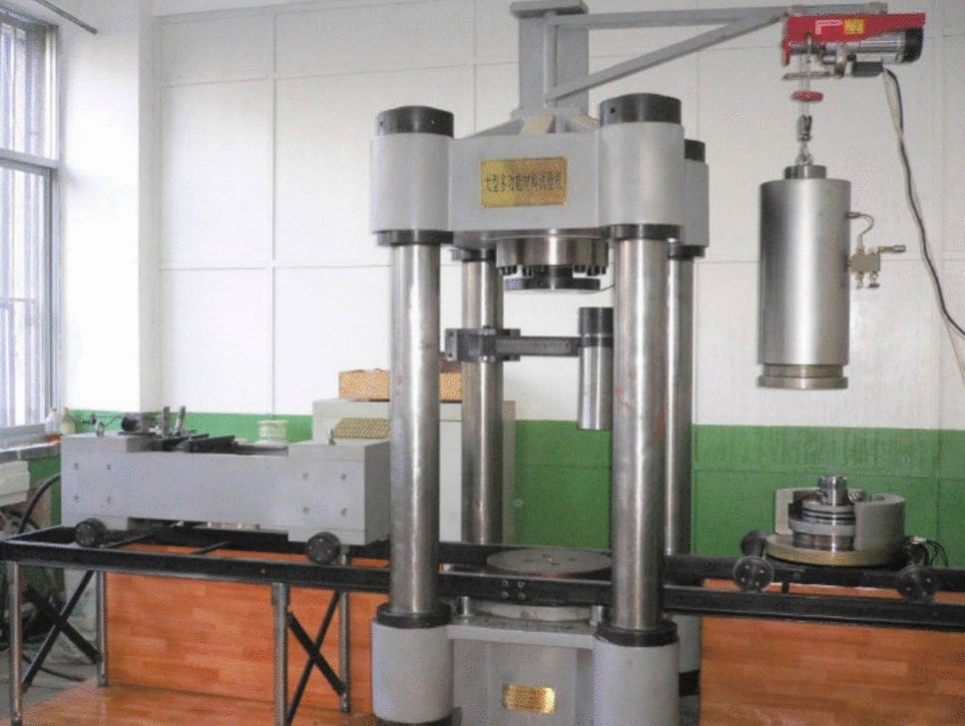
WDT-1500 multi-purpose material testing machine.

The triaxial cyclic tests in this study were conducted using the WDT-1500 multifunctional material testing machine. This device was jointly developed by the Geotechnical Institute of Xi’an University of Technology and Changchun Chaoyang Testing Instruments Co., Ltd, as shown in Fig. 2. The control system adopts DOLI fully digital servo controller imported from Germany. The WDT-1500 is a rigid servo dynamic mechanical testing device designed primarily for high-strength materials such as rock and concrete. It is characterized by its versatility, high precision, excellent stability, ease of operation and safety.

The WDT-1500 multifunctional material testing machine is capable of performing various types of tests, including uniaxial compression, shear strength, fatigue and creep tests. It can flawlessly execute the experimental procedures set forth in this study, offering high precision, strong resistance to interference and minimizing errors caused by environmental factors. The entire process is controlled by personal computer (PC). A high safety factor and reducing potential risks are ensured.

**Fig. 3:**
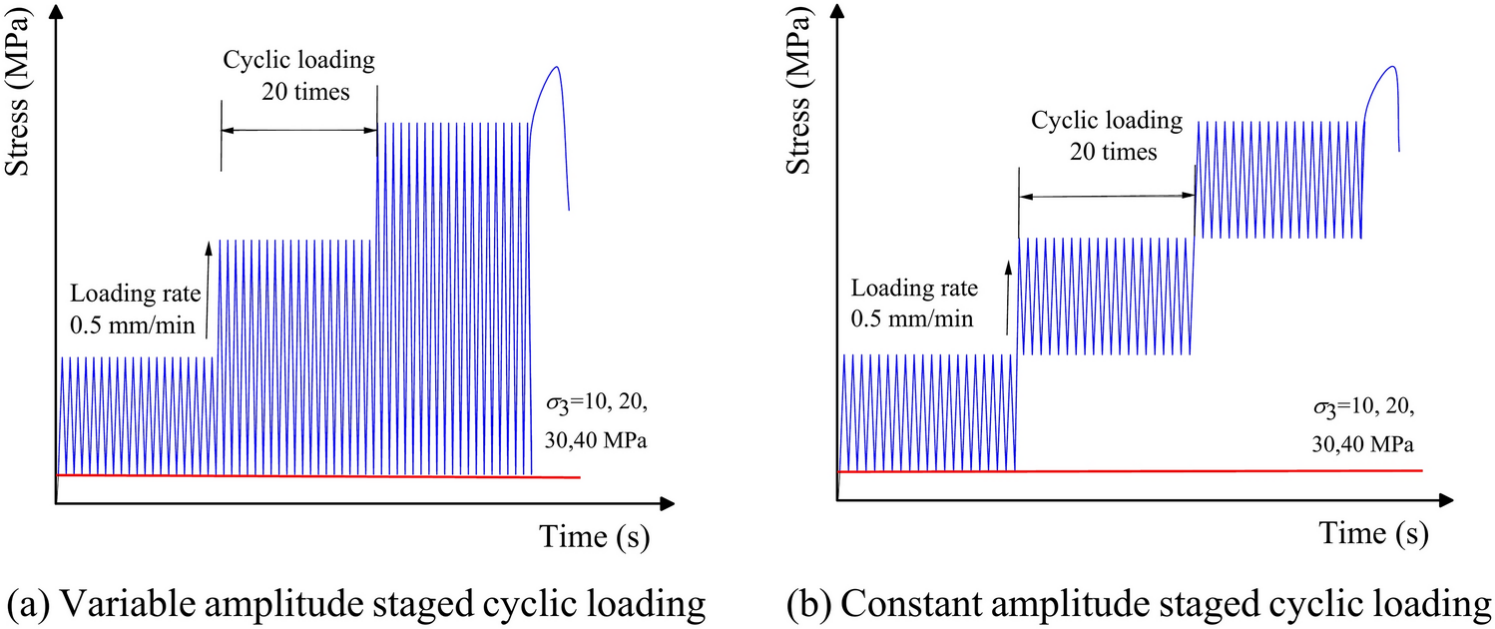
Schematic diagram of cyclic loading and unloading path.

This study is divided into two types of cyclic loading tests with different loading methods: amplitude-variable staged cyclic loading tests and constant amplitude staged cyclic loading tests. The schematic diagram of the cyclic loading and unloading path is shown in Fig. 3. The concepts of stress ratio and strain ratio are introduced in this study to facilitate the analysis of different rock types at the same stage. The stress ratio *i*_*1*_ is defined as the ratio of the dynamic maximum stress *σ*_*max*_ to the peak stress *σ*_*c*_, and the strain ratio *i*_*0*_ is defined as the ratio of the dynamic maximum strain *ε*_*max*_ to the peak strain *ε*_c_. This approach prevents erroneous comparisons between the elastic stage of one rock and the yield stage of another, thereby enabling a more accurate analysis of the experimental phenomena. The loading rate for both staged cyclic loading tests is set at 0.5 mm/min, with a frequency of 0.2 Hz.

The specimen is first subjected to the specified confining pressure in the amplitude variable staged cyclic loading test. Then cyclic loading is applied to the specimen. The strain upper limit is increased through the testing machine after 20 cycles in the first stage. Meanwhile, the strain lower limit remains unchanged. This process is repeated by further increasing the strain upper limit, while keeping the lower limit constant. The test is conducted until the specimen fails. A sudden and steep decline in the peak stress of the stress–strain curve indicates that the specimen has reached a state of failure. The experimental scheme of the variable amplitude staged cyclic loading experiment is shown in Table 1.

**Table 1:** The test scheme of graded cyclic loading with different amplitude.

Rock types	Confining pressure (MPa)	Strain upper limit	Strain lower limit	Upper limit increment	Lower limit increment
Sandstone	5	0.5	0	0.1	0
	10				
	15				
	20				
	25				
Granite	5	0.1	0	0.1	0
	10				
	15				
	20				
	25				

The specimen is also initially subjected to the specified confining pressure in the constant amplitude staged cyclic loading test. After 20 cycles in the first stage, both the strain upper and lower limits are increased by the same amount to move to the next stage. This procedure continues with simultaneous adjustments to both the upper and lower strain limits. Cyclic loading is applied 20 times at each stage until the specimen fails. The experimental scheme of the constant amplitude staged cyclic loading experiment is shown in Table 2.

**Table 2:** The test scheme of graded cyclic loading with the same amplitude.

Rock types	Confining pressure (MPa)	Strain upper limit	Strain lower limit	Upper limit increment	Lower limit increment
Sandstone	5	0.5	0	0.1	0.1
	10				
	15				
	20				
	25				
Granite	5	0.1	0	0.1	0.1
	10				
	15				
	20				
	25				

## Energy model

The physical state of a system is reflected by the energy transformations of the system according to the laws of thermodynamics. There is a certain relationship between the instability of the system and the changes in energy. Various strain amplitudes, stress levels, confining pressures, and cycle counts in the loading and unloading tests of sandstone and granite are analyzed from an energy perspective. This can explore the changing laws of rock energy during the test and analyze the principles behind it. The input energy is converted into elastic energy, plastic energy, dissipated energy and thermal energy. Since the experiments are conducted at constant temperature, thermal energy dissipation is neglected. Plastic energy is associated with plastic deformation in the system. Elastic energy is stored within the system during the loading phase. This portion of deformation is fully recovered and the elastic energy is completely released upon unloading. Dissipated energy arises from factors such as crack propagation in the specimen and the friction between mineral particles. The variation in dissipated energy is investigated with great significance.

The accumulation of plastic deformation and damage causes the residual strain upon unloading to exceed the initial strain at the onset of loading under cyclic loading, forming a closed loop in the stress–strain curve, known as the hysteresis loop. This phenomenon reflects the energy dissipation and irreversible deformation of the rock during the loading and unloading process. The hysteresis loop formed during cyclic loading is a manifestation of energy dissipation. The size of the hysteresis loop can be used to describe the magnitude of dissipated energy. The area of the hysteresis loop represents the energy dissipated by the rock in one cycle. The calculation formula for dissipated energy *U*^*d*^ can be expressed as:

1 $$U^{d} = \int_{{\varepsilon_{min} }}^{{\varepsilon_{max} }} {\sigma d\varepsilon - } \int_{{\varepsilon_{{_{max} }} }}^{{\varepsilon_{{_{min} }} }} {\sigma d\varepsilon } = A_{1}$$

where A_1_ is the area of the hysteresis loop.

**Fig. 4:**
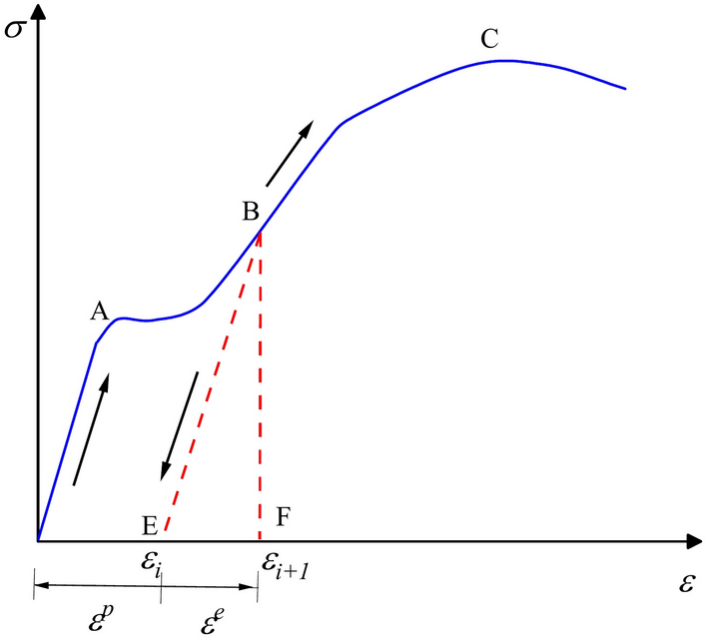
Loading and unloading stress–strain curve.

**Fig. 5:**
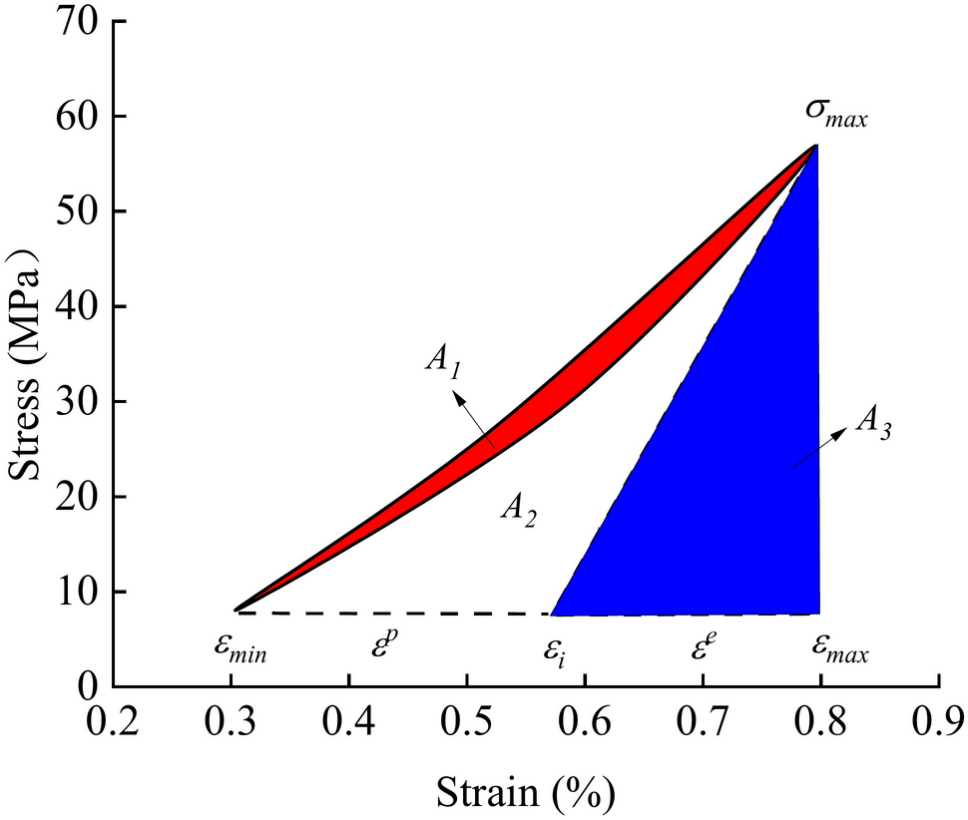
Energy relationship diagram under cyclic loading.

Figure 4 is a schematic diagram of the loading and unloading stress–strain curve. It reflects the elastic–plastic behavior of the material during loading and unloading. Points A, B, and C correspond to the elastic limit, yield point, and ultimate strength, respectively. Segments E–F represent the unloading process. Elastic energy refers to the recoverable deformation energy stored within the system. A portion of the deformation generated by the system will be fully recovered upon stress unloading during stress loading. This characteristic is represented by a straight line on the stress–strain curve as shown in Fig. 4. The elastic portion typically appears as a linear or nearly linear region in stress–strain curve. The elastic energy can be obtained by calculating the area of the elastic portion of the stress–strain curve. Figure 5 is the energy relationship diagram under cyclic load. Based on Fig. 4 and Fig. 5, it is assumed that the magnitude of elastic energy in the system corresponds to the area of triangle A_3_. By calculating the slope *K* of the unloading segment, the peak stress, peak strain, and slope *K* can be substituted into the equation to derive the equation of the straight line for the elastic segment’s hypotenuse in the coordinate system.

2 $${\text{y}} = Kx + \sigma_{{{\text{max}}}} - K\varepsilon_{{{\text{max}}}}$$

where *K* is the slope of the unloading segment. *σ*_*max*_ represents the peak stress, while *ε*_*max*_ corresponds to the peak strain.

Substituting the minimum strain coordinates of the hysteresis loop as shown in Fig. 5, the *ε*_*i*_ can be obtained as:

3 $$\varepsilon_{{\text{i}}} = \varepsilon_{{{\text{max}}}} + \frac{{\sigma_{{{\text{min}}}} { - }\sigma_{{{\text{max}}}} }}{k}$$

where, *ε*_*i*_ represents the critical point of plastic strain, while *σ*_*min*_ denotes the minimum stress of the hysteresis loop. *k* is the stiffness coefficient.

The elastic energy *U*^*e*^ at this point can be expressed as:

4 $$U^{{\text{e}}} = A_{3} = \frac{{(\varepsilon_{{{\text{max}}}} - \varepsilon_{i} ) \cdot (\sigma_{\max } - \sigma_{{{\text{min}}}} )}}{2}$$

Equations (3) and (4) are combined to obtain the plastic performance calculation formula:

5 $$U^{{\text{e}}} = \frac{{(\sigma_{\max } - \sigma_{{{\text{min}}}} )^{2} }}{2k}$$

The portion representing plastic energy in Fig. 5 is the area of region A_2_. The expression for plastic energy *U*^*p*^ is:

6 $$U^{p} = U - U^{e} - U^{d}$$

where, *U* represents the total work done by the system, which is the area enclosed by the loading segment curve and the coordinate axes.

By organizing the experimental curves and applying the above formulas, the dissipated energy *U*^*d*^, elastic energy *U*^*e*^ and plastic energy *U*^*p*^ of the system are calculated. The influence of different variables on each energy parameter is analyzed to identify underlying patterns, which are then examined and explained.

## The influence of different strain amplitudes on energy variation

Sandstone is composed of sand-sized particles bound by cementing materials. This bonding is not tight, resulting in the presence of voids inside the sandstone. The original pores are compacted first under load conditions. The deformation of the weak cementing substances follows. The cracks begin to expand until they connect with each other, eventually leading to the failure of rock. The compaction of these voids, the deformation of the cementing materials and the expansion of cracks all contribute to energy dissipation. This dissipation reflects the development of internal defects within the rock and the gradual reduction in material strength. This section analyzes and summarizes the variations in dissipated energy under different strain amplitudes and loading conditions. The reasons behind the observed patterns are explained.

### Dissipated energy

**Fig. 6:**
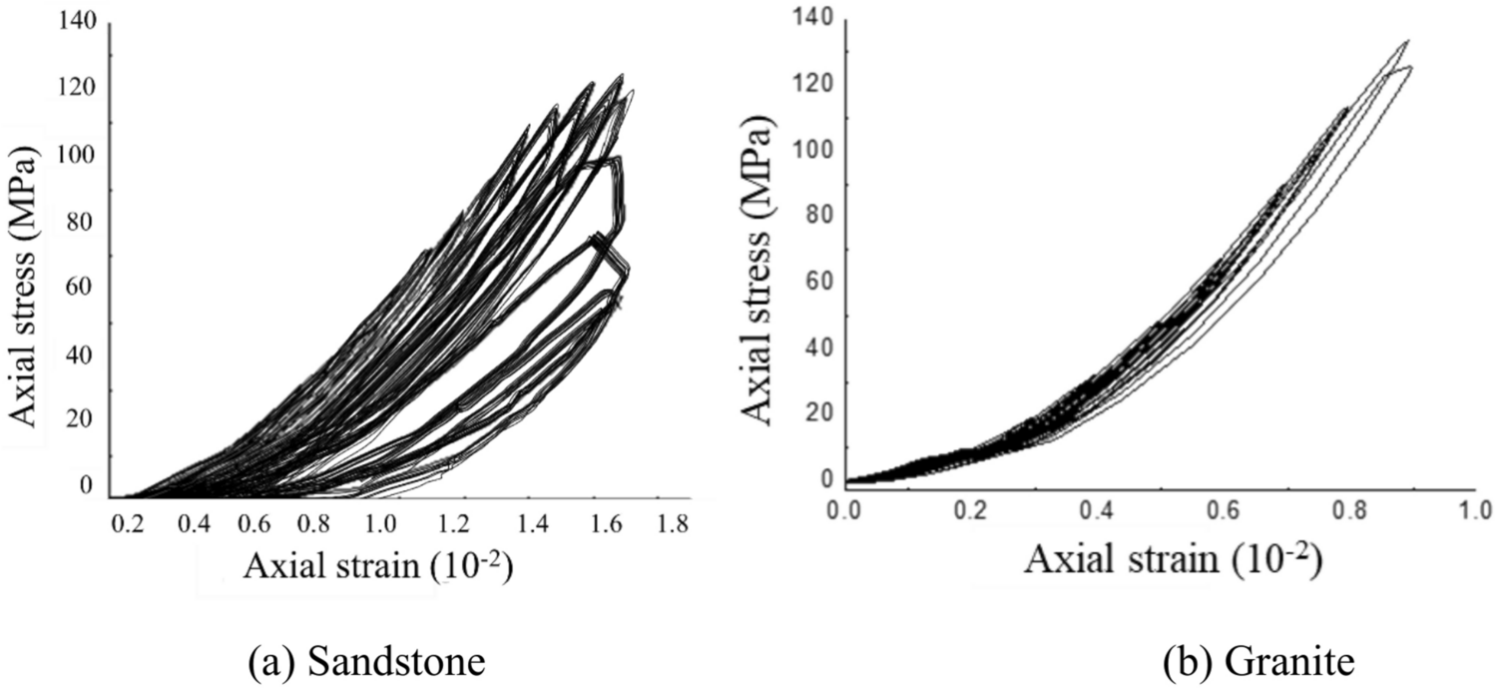
Typical stress–strain curves of graded cyclic loading tests with different strain amplitudes.

Data with varying strain amplitudes were analyzed and organized in the completed cyclic loading tests. The experimental curves of sandstone and granite under different strain amplitude staged cyclic loading conditions are shown in Fig. 6. The dissipated energy for both sandstone and granite was calculated across the entire test cycle. The data was processed to obtain the variation curves of dissipated energy. The total energy input *U* during the experiment is converted into recoverable elastic strain energy *U*^*e*^, dissipated energy *U*^*d*^ and plastic energy *U*^*p*^. This section focuses on the analysis of dissipated energy *U*^*d*^.

Sandstone has a high porosity and is prone to deformation during loading. Energy dissipation is mainly reflected in the compression and closure of pores and the relative slip of local particles. Granite has a low porosity and energy dissipation is mainly through crack extension and inter-grain slip, so porosity has little effect on dissipated energy. Sandstone has a high crack density due to diagenesis and weathering. More energy is used for crack extension during loading, resulting in higher dissipated energy. Granite is more prone to brittle fracture under low crack density, resulting in lower dissipated energy. Sandstone has a low degree of cementation and weak inter-particle bonding. Particle sliding and rearrangement during loading will increase energy dissipation. Granite has a high degree of cementation and tightly bonded particles. Energy dissipation mainly comes from crack extension, and the degree of cementation has little effect on energy dissipation.

**Fig. 7:**
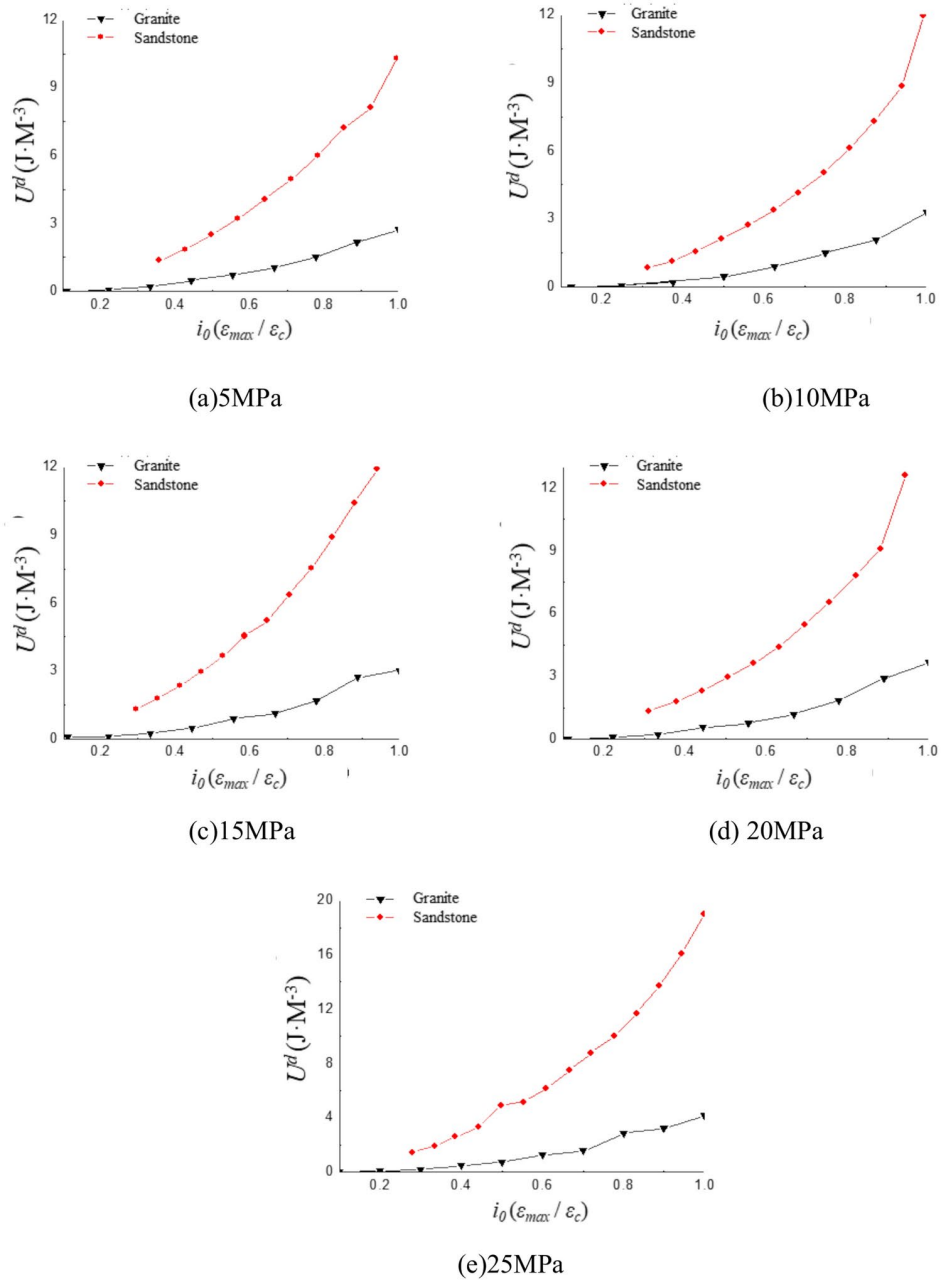
Variation curve of dissipation energy under different confining pressures.

To facilitate a better comparison between different rock types, the strain ratio *i*_*0*_ (defined as the dynamic maximum strain *ε*_*max*_ / the peak strain *ε*_*c*_) is derived. Sandstone and granite have different mechanical properties and behaviors under dynamic loading conditions. Sandstone is more ductile and can withstand larger deformations before failure. Therefore, the strain ratio for sandstone starts at 0.3, indicating that it can withstand a wider range of strains before failure, especially under dynamic loading conditions. Granite is more brittle and tends to fail at lower strain levels. Therefore, the strain ratio for granite starts at 0.1, reflecting its more limited ability to deform before rupture or severe damage under cyclic loading. The curve of dissipated energy as a function of strain ratio *i*_*0*_ is shown in Fig. 7. The dissipated energy for both sandstone and granite increases gradually with the strain ratio. The rate of increase in dissipated energy becomes progressively larger as the strain amplitude increases. However, there is a distinct difference between granite and sandstone. The variation in dissipated energy is relatively gradual for granite, whereas it is more pronounced for sandstone. The dissipated energy increases with strain amplitude at a higher rate in sandstone than in granite under the same strain ratio.

**Fig. 8:**
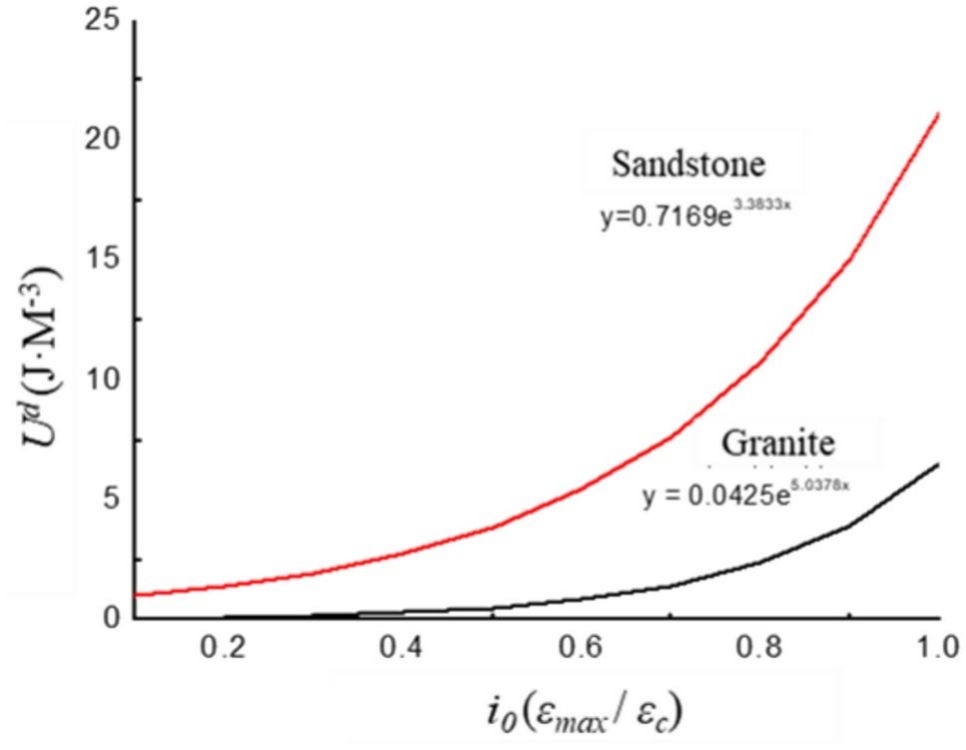
Fitting curve of dissipation energy with strain ratio.

The fitted curves for the variations in dissipated energy for sandstone and granite are presented in Fig. 8. It is evident that dissipated energy for both sandstone and granite follows an exponential function trend as the strain ratio increases. The exponential function fitted to the curves at a confining pressure of 5 MPa is labeled in Fig. 8. The rate of increase in dissipated energy for sandstone is significantly higher than that for granite. The difference in dissipated energy between sandstone and granite becomes more pronounced as the strain ratio increases.

This phenomenon can be explained by the process of energy dissipation within the rock. It is assumed that there is no heat loss in the entire experimental system. The energy dissipation of the specimen primarily depends on internal work. The deformation is mainly elastic and can be recovered when the strain is small. At this stage, the energy dissipation is low, and the rate of increase is gradual. As the strain amplitude increases, crack propagation intensifies, and the energy input into the system is primarily devoted to crack expansion. It results in a gradual increase in energy dissipation and the rate of energy increase rises sharply.

The crack propagation of sandstone starts at the contact surface between particles or due to stress concentration in the pores. When stress is applied, microcracks will be generated at the interface between particles, and these microcracks will gradually develop into macro cracks with the repeated action of cyclic stress. During the loading and unloading process of sandstone, the dissipated energy is mainly caused by the generation of cracks and particle sliding^26^. The crack propagation of granite is related to the aggregation, connection and propagation of microcracks. The energy mechanism of crack propagation is mainly manifested as the release of elastic energy and the fracture of grain interfaces during the cyclic loading and unloading process of granite. During the loading process, granite accumulates strain energy, and during the unloading process, the accumulated energy is released, resulting in crack propagation^26^. During the loading and unloading cycle, each loading will increase the stress field at the crack tip, causing the crack to gradually expand. When unloading, part of the energy is released, and the remaining energy is stored in the elastic deformation of the rock^53^.

The faster rate of change in dissipated energy with respect to amplitude in sandstone compared to granite under the same strain ratio. The differing lithologies of the two rocks. Sandstone is a sedimentary rock with a relatively loose structure. Granite is an igneous rock with a much denser structure compared to sandstone. The primary source of dissipated energy is the friction, deformation, and propagation of defects such as cracks within the rock structure. Sandstone contains far more defects than the denser granite due to its relatively loose composition. Sandstone is more prone to energy dissipation than granite as the strain amplitude increases.

### Elastic energy

**Fig. 9:**
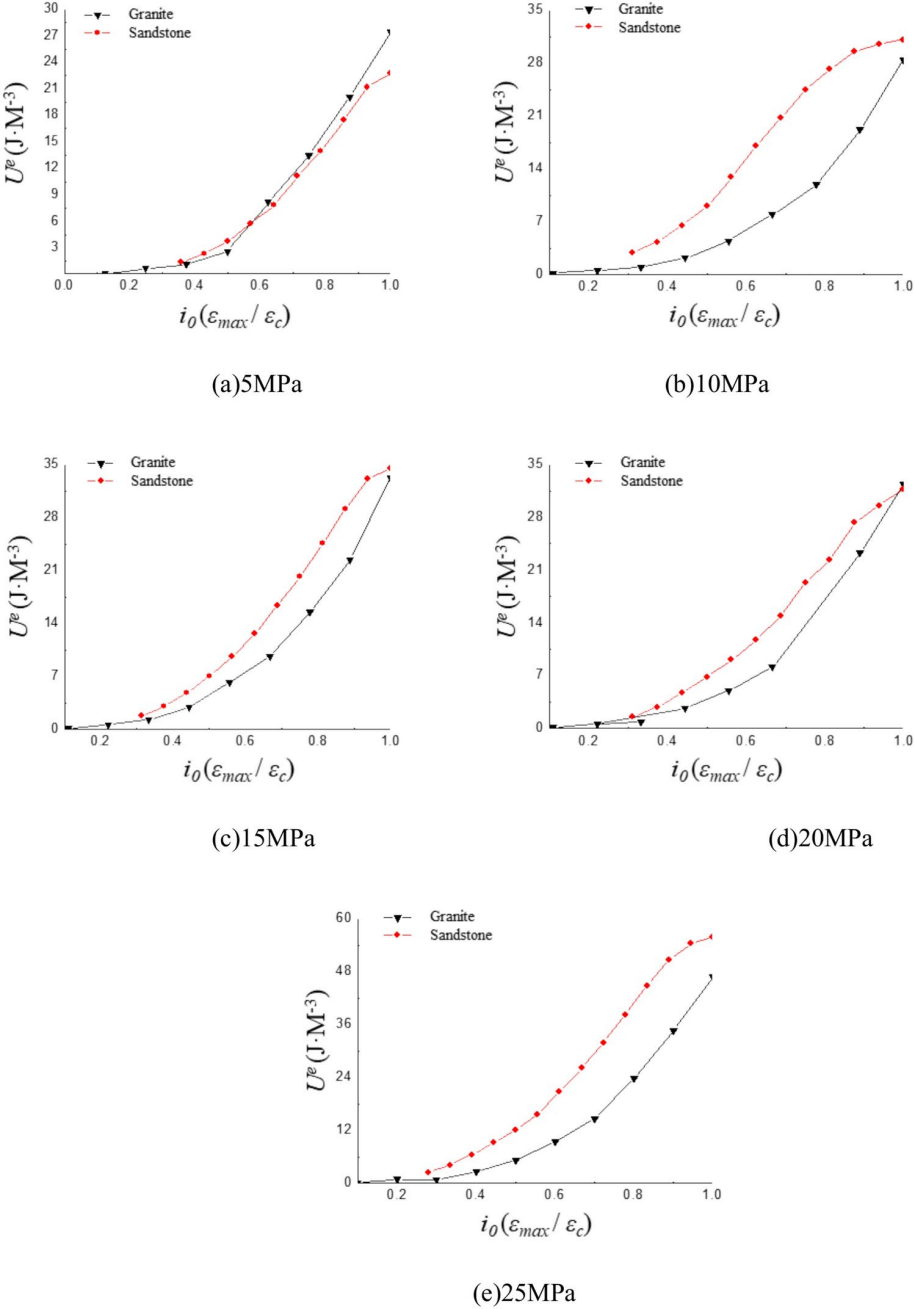
Curve of the variation of the elastic energy and the accompanying amplitude under different confining pressure.

Different strain amplitudes result in variations in the energy output throughout the entire experimental process. The curves are plotted to demonstrate how elastic energy varies with different strain amplitudes. The variation pattern is then analyzed and explained. The relationship between elastic energy and strain amplitude is shown in Fig. 9.

As shown in Fig. 8, the elastic energy generally shows an increasing trend with the increase in strain amplitude. The rate of increase varies with different strain ratios, and distinct trends are observed between sandstone and granite. The specific details are shown in Table 3:

**Table 3:** Elastic energy changes with strain ratio rate table under different confining pressures.

Rock types	*i*_*0*_	Confining pressure (MPa)				
		5	10	15	20	25
Sandstone	0.1	4.58	3.37	3.97	3.59	4.34
	0.2	3.99	3.58	4.91	3.16	3.58
	0.3	7.71	7.15	10.37	9.45	9.13
	0.4	28.15	15.64	22.09	18.63	22.23
	0.5	43.57	26.19	30.40	24.39	34.07
	0.6	47.78	33.52	42.04	48.03	46.59
	0.7	55.59	50.45	57.30	75.25	71.65
	0.8	58.48	74.52	80.02	82.11	100.61
Granite	0.3	13.27	22.65	20.43	21.22	30.87
	0.4	16.27	29.01	24.21	25.78	36.73
	0.5	23.94	38.27	32.15	31.96	44.96
	0.6	28.85	51.01	38.34	35.04	50.89
	0.7	37.36	63.48	44.57	39.48	58.52
	0.8	42.57	62.61	54.30	46.62	77.01
	0.9	44.38	59.92	60.46	60.68	94.18
	1	50.87	51.39	65.62	58.97	101.17

Granite can be divided into three stages under different confining pressures. The first stage occurs when the strain ratio is between 0.1 and 0.2. The rate of increase in elastic energy with respect to the strain ratio is slow during this phase. The second stage occurs when the strain ratio is between 0.2 and 0.8. The rate of increase in elastic energy accelerates significantly in this phase. The third stage begins when the strain ratio exceeds 0.8 at which point the growth trend of elastic energy stabilizes. Similarly, sandstone can be divided into three stages based on the strain ratio: 0.3 to 0.4, 0.4 to 0.8, and 0.8 to 1. In all three stages, the growth rate of elastic energy initially increases slowly, then accelerates. Once the strain ratio exceeds 0.8, the growth rate once again slows down.

The elastic deformation generated by the specimen is also relatively minor when the strain is relatively small. The elastic energy generated by specimen is not fully activated by the corresponding deformation. Specimen is still capable of generating a significant amount of elastic energy at this point. The increase in elastic energy occurs at a slower rate at lower strain ratios. The specimen continues to display strong elastic characteristics as the strain ratio increases, remaining within 40% to 80% of the peak strain. The deformation of the specimen gradually becomes more pronounced with further increase in strain ratio. The recoverable deformation reaches its maximum upon failure.

### Plasticity energy

**Fig. 10:**
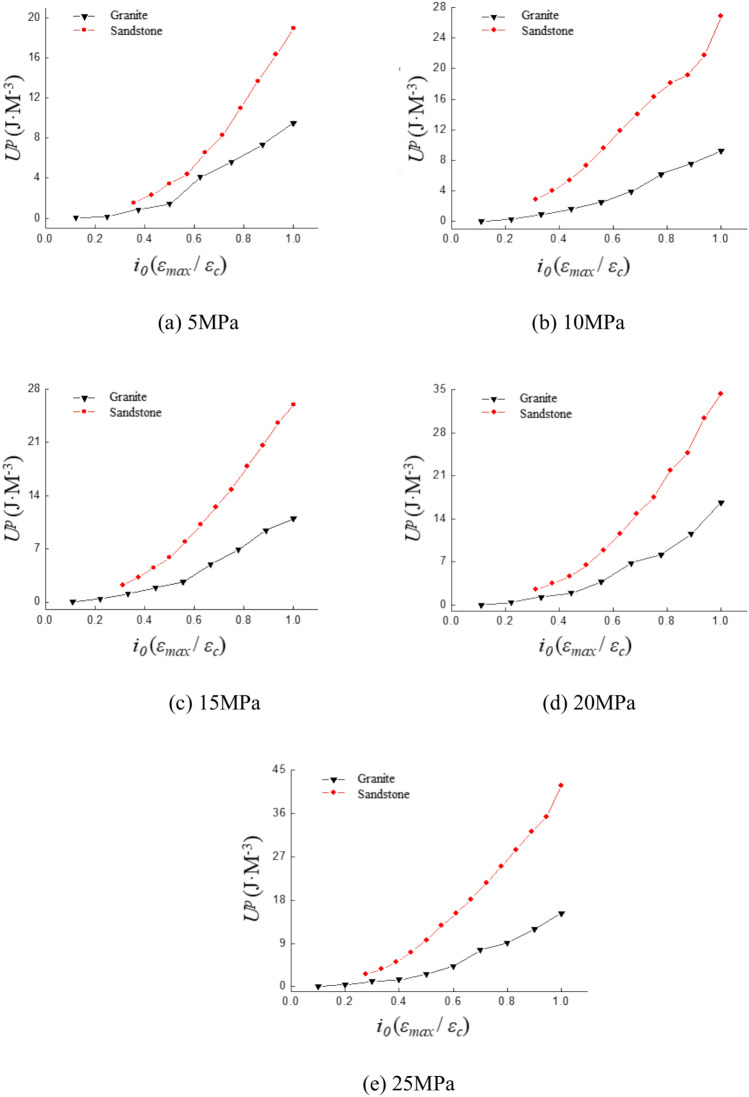
Curve of plastic properties with strain ratio under different confining pressures.

The variation patterns of dissipated energy and elastic energy during the cyclic loading process are analyzed. The variation of plastic energy with increasing strain amplitude is also studied. The plastic energy variation curves are shown in Fig. 10. The results indicate that the plastic energy of both sandstone and granite gradually increases with the strain ratio. Plastic energy exhibits a consistent growth trend with increasing strain ratio under different confining pressures.

The variation pattern of plastic energy with strain amplitude remains generally consistent under different confining pressures as shown in Fig. 10. The fitted equations obtained by fitting the curves for the same lithology are summarized in the table below:

Table 4 reveals that plastic energy exhibits an exponential increase with growing strain amplitude, with variation trends differing under different confining pressures. The rate and magnitude of plastic energy increase in sandstone surpass those observed in granite. This variation trend can be further analyzed by exploring the mechanisms underlying plastic energy.

**Table 4:** The fitting equation for the variation of plastic Properties with strain amplitude.

Confining pressure (MPa)	Fitting equation	
	Sandstone	Granite
5	y = 0.4493e^2.0182x^	y = 0.06790e^5.538x^
10	y = 1.3340e^2.0039x^	y = 0.0675e^5.6415x^
15	y = 0.8640e^2.3219x^	y = 0.1158e^5.1413x^
20	y = 0.9127e^2.3898x^	y = 0.0990e^5.7015x^
25	y = 1.2818e^2.0628x^	y = 0.1355e^5.2655x^

Plastic energy is the result of plastic deformation, which is an irreversible form of deformation. Specimen remains in the elastic phase with relatively small strain amplitudes at low strain ratios. Elastic deformation constitutes the majority of the total strain during this phase. The specimen transitions from the elastic phase to the yield phase as the strain ratio increases. This leads to a gradual increase in both plastic deformation and plastic energy. Cracks propagate instantaneously and a sharp rise in irreversible deformation is caused at the point of failure. The overall variation curve aligns closely with the trend of an exponential function.

## Effect of same strain amplitude on energy variation

This section primarily investigates and discusses the variation patterns of dissipative energy, elastic energy and plastic energy under the same strain amplitude graded cyclic loading. The influence of various factors on these energies is analyzed, along with the underlying causes of these effects. For ease of comparison, the stress ratio *i*_*1*_ (defined as the ratio of the dynamic maximum stress *σ*_*max*_ / the peak stress *σ*_*c*_) is used as the variable for analysis.

### Dissipated energy

**Fig. 11:**
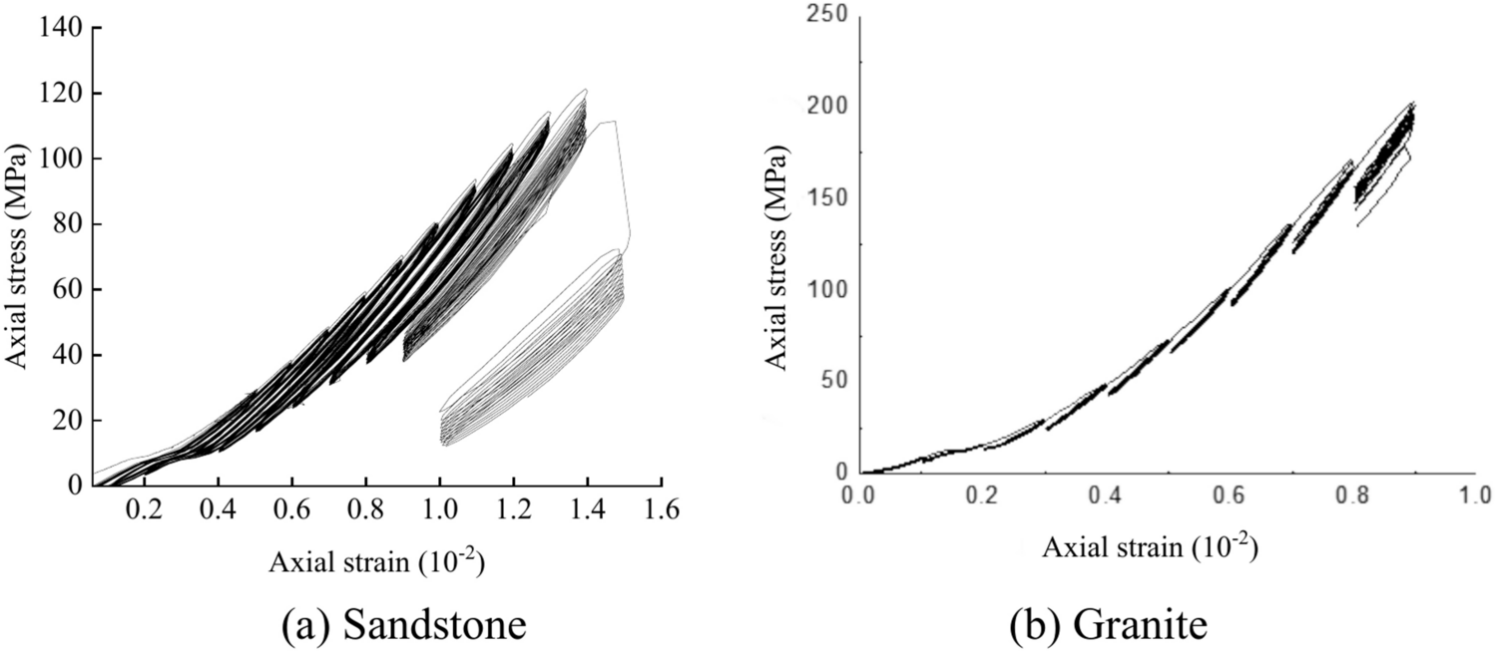
Stress–strain curve of the same strain amplitude staged loading test.

To discuss the variation patterns of dissipative energy under different stress levels, the cyclic loading test data with the same amplitude were organized. The experimental curves are shown in Fig. 11. Sandstone is mainly composed of quartz, feldspar and cement, with high porosity and loose structure. There are many pores and cracks inside the sandstone. The pores are first compressed, then partially rebound during unloading and finally irreversible damage is accumulated during the stress cycle. Sandstone is prone to shear failure under high confining pressure, which is mainly manifested by the gradual expansion of shear cracks and local particle crushing. Granite is primarily composed of minerals such as quartz, feldspar, and mica, with relatively few internal microcracks. The initial fractures are primarily influenced by high-stress concentrations. These fractures rapidly propagate during stress cycling, ultimately leading to brittle fracture.

**Fig. 12:**
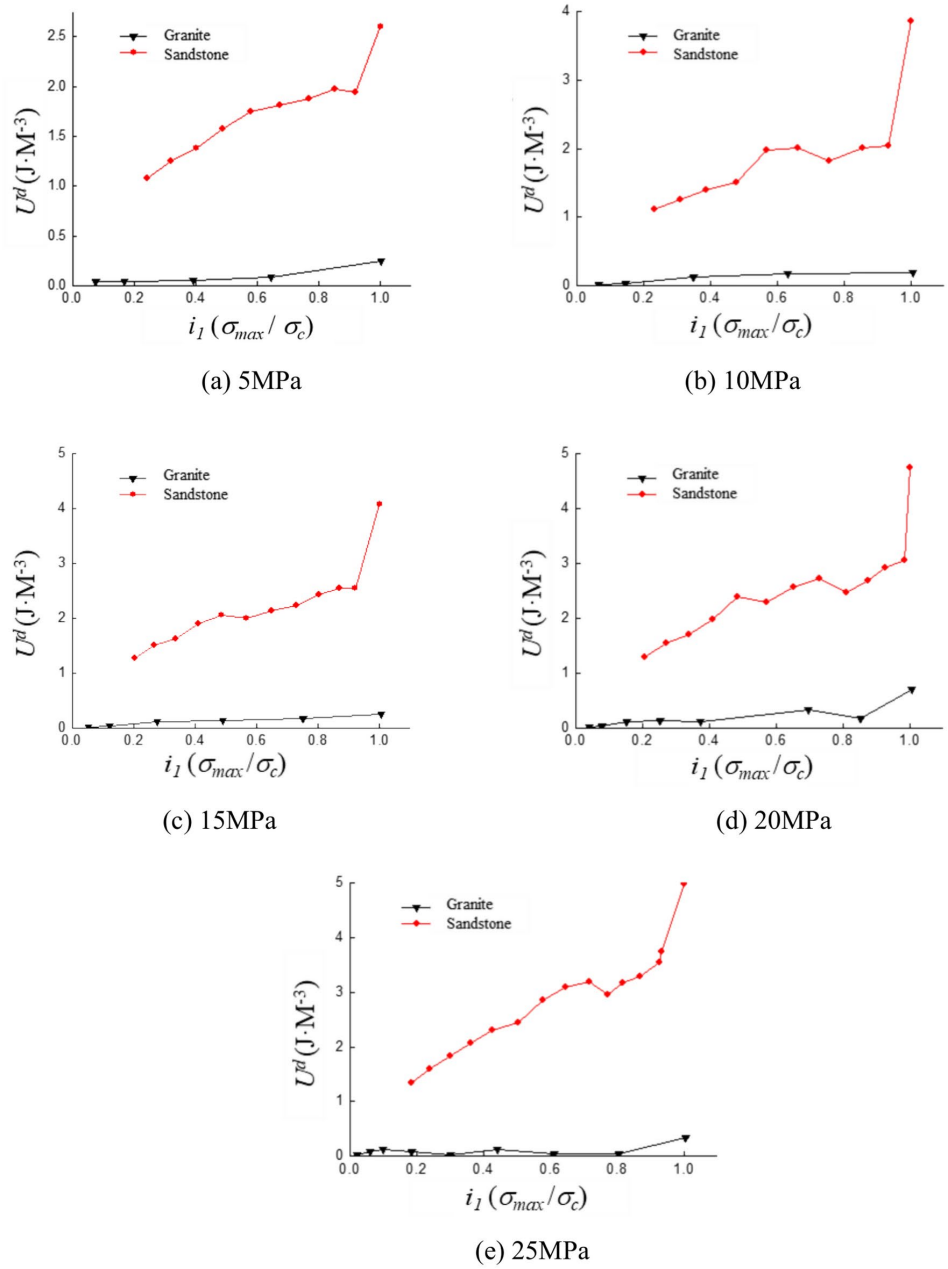
Curves of energy dissipation with stress level under different confining pressure.

As shown in Fig. 12, dissipation energy increases with the rise in stress level under the same confining pressure and strain amplitude. The rate of increase varies with different confining pressures. The slope of the curve for sandstone changes around a stress ratio of 0.85 in Fig. 12a. The change rate of dissipation energy with respect to stress ratio is 1.31 when the stress ratio is less than 0.85. The strain ratio increases by a factor of 6.15 as the stress level increases. The slope of the dissipation energy curve for sandstone increases at a stress ratio of 0.91 in Fig. 12b. When the stress ratio exceeds 0.9, the slope increases by a factor of 17.38. In Fig. 12c, the dissipation energy curve for sandstone changes at a stress ratio of 0.90 with the slope increasing by a factor of 11.3. In Fig. 12d, the curve changes at a stress ratio of 0.95 and the slope increases by 12.1 times. In Fig. 12e, the slope changes at a stress ratio of 0.94 with an increase of 7.35 times. The increase in dissipation energy for sandstone between stress ratios of 0.85 and 0.95 is significantly greater than before.

The dissipated energy presents three stages with the stress ratio, first rising, then fluctuating slightly, and finally showing a steep rising trend. The dissipated energy represents the amount of internal energy loss of rock during deformation and destruction. The rock is in the elastic deformation stage and the energy is stored in the form of elastic energy when the stress is relatively low, resulting in lower energy dissipation. After entering the plastic deformation stage, irreversible deformations such as microcracks and inter-particle slip begin to occur inside the rock, resulting in a slow increase in dissipated energy. When the stress ratio approaches the limit, cracks rapidly propagate and connect, leading to rock failure. This process is accompanied by a violent release of energy, which causes the dissipated energy to rise sharply and form a steep upward trend.

The variation trend in granite is less pronounced compared to sandstone. The rate of increase in dissipative energy for granite exhibits a sudden change as the stress ratio approaches the failure stage. The explanation for this phenomenon is as follows:

The failure of the specimen is closely related to its strength state during the cyclic loading process. The energy behavior during the experiment primarily manifests as energy release and dissipation. As axial plastic deformation occurs and primary and secondary fractures propagate, a significant amount of dissipated energy is consumed. When the stress ratio is between 0.85 and 0.95, the specimen enters the strengthening stage and its strength approaches the peak value.

The energy dissipation process is mainly attributed to the expansion and percolation of fractures. Once the internal energy accumulates to a certain threshold, the main fracture gradually connects. The displacement and friction between fractures cause a sudden increase in dissipative energy. The onset of failure is indicated. The abrupt rise in dissipative energy can also serve as an indicator for assessing the failure of rock strength. There is also a critical point for crack expansion, which is manifested as a sharp increase in dissipated energy. Energy dissipation is mainly manifested as local plastic deformation and the formation of microcracks in the crack initiation stage. In the crack initiation stage, the initial cracks in the rock gradually close due to the low stress levels, preventing the formation of new cracks. Energy dissipation is mainly manifested in local plastic deformation and the formation of microcracks. The process of closing the initial crack requires consumption of dissipated energy, so the rate of increase of dissipated energy is relatively fast. The stress exceeds the rock cracking stress during the crack extension stage, causing new cracks inside the rock to begin to initiate and expand and the dissipated energy increases slowly. The cracks inside the rock are connected in large numbers and form macroscopic fracture surfaces when the cracks reach the critical point leading to rock destruction. A large amount of energy is dissipated in an irreversible way, and the dissipated energy increases rapidly.

### Elastic energy

**Fig. 13:**
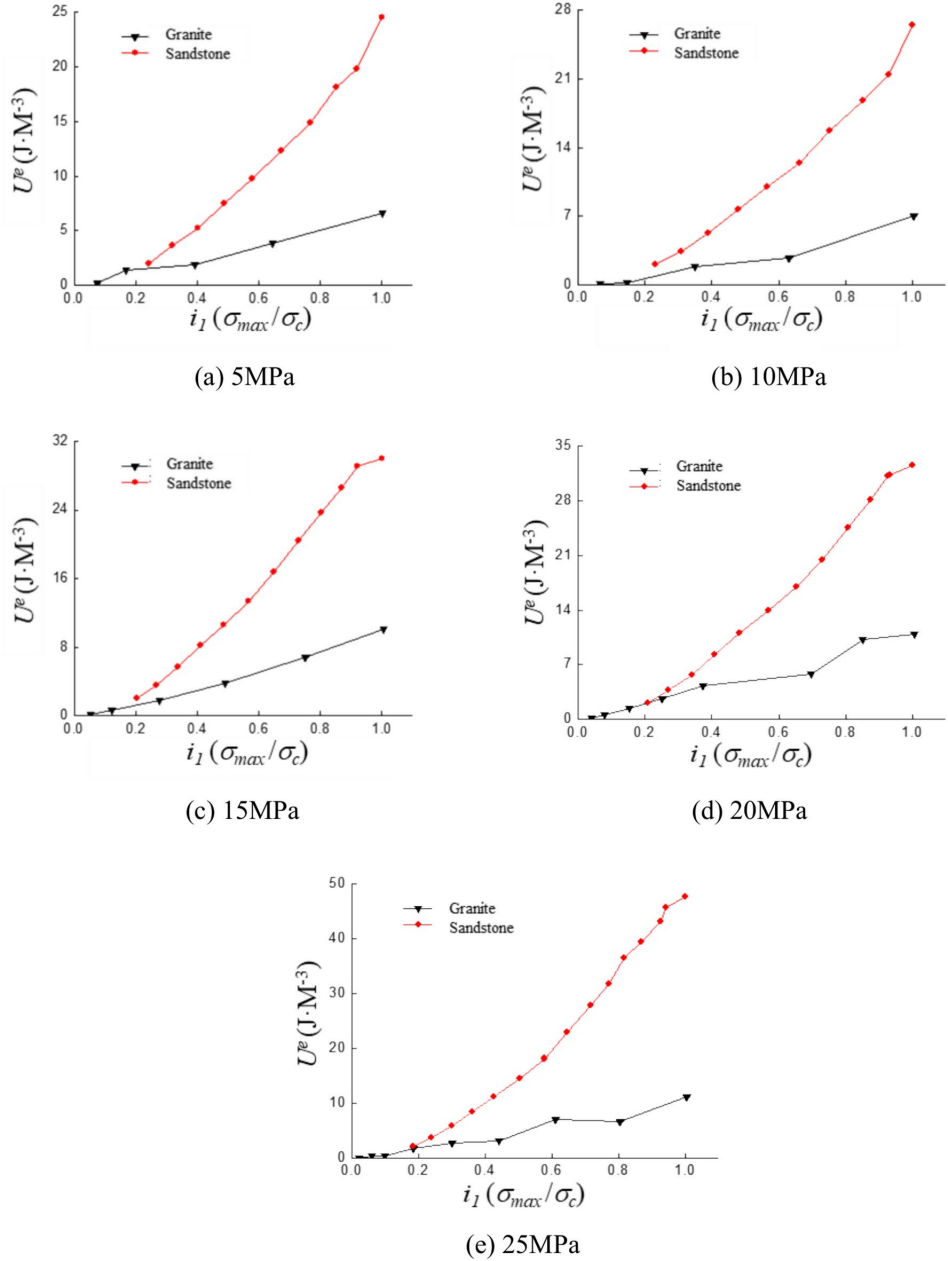
Change curve of elastic properties under graded loading with the same amplitude.

The overall trend of elastic energy variation in sandstone and granite shows that the elastic energy gradually increases with the increase of the stress ratio. As shown in Fig. 13a, it is evident that the elastic energy of sandstone increases with the stress ratio. The rate of increase also progressively accelerates. An exponential function characteristic is exhibited. The curve for granite increases linearly with a relatively stable rate of increase and a smaller amplitude of change. Further analysis of Fig. 13b–e reveals that their trends are generally consistent with the elastic energy variation observed at confining pressure of 5 MPa. Therefore, it can be concluded that with the increase in stress ratio, both sandstone and granite exhibit an increase in elastic energy.

The elastic deformation of sample is the mechanism through which elastic energy is generated. The increase in stress ratio under the same strain amplitude means that both the upper and lower limits of stress increase during the cycle. This elevation causes the sample’s state to transition from the initial compaction phase to the elastic phase. The elastic energy of the sample gradually increases. The elastic energy of sandstone increases faster than that of granite. This indicates that sandstone exhibits stronger elastic properties compared to granite with an increase in the stress ratio.

### Plasticity energy

**Fig. 14:**
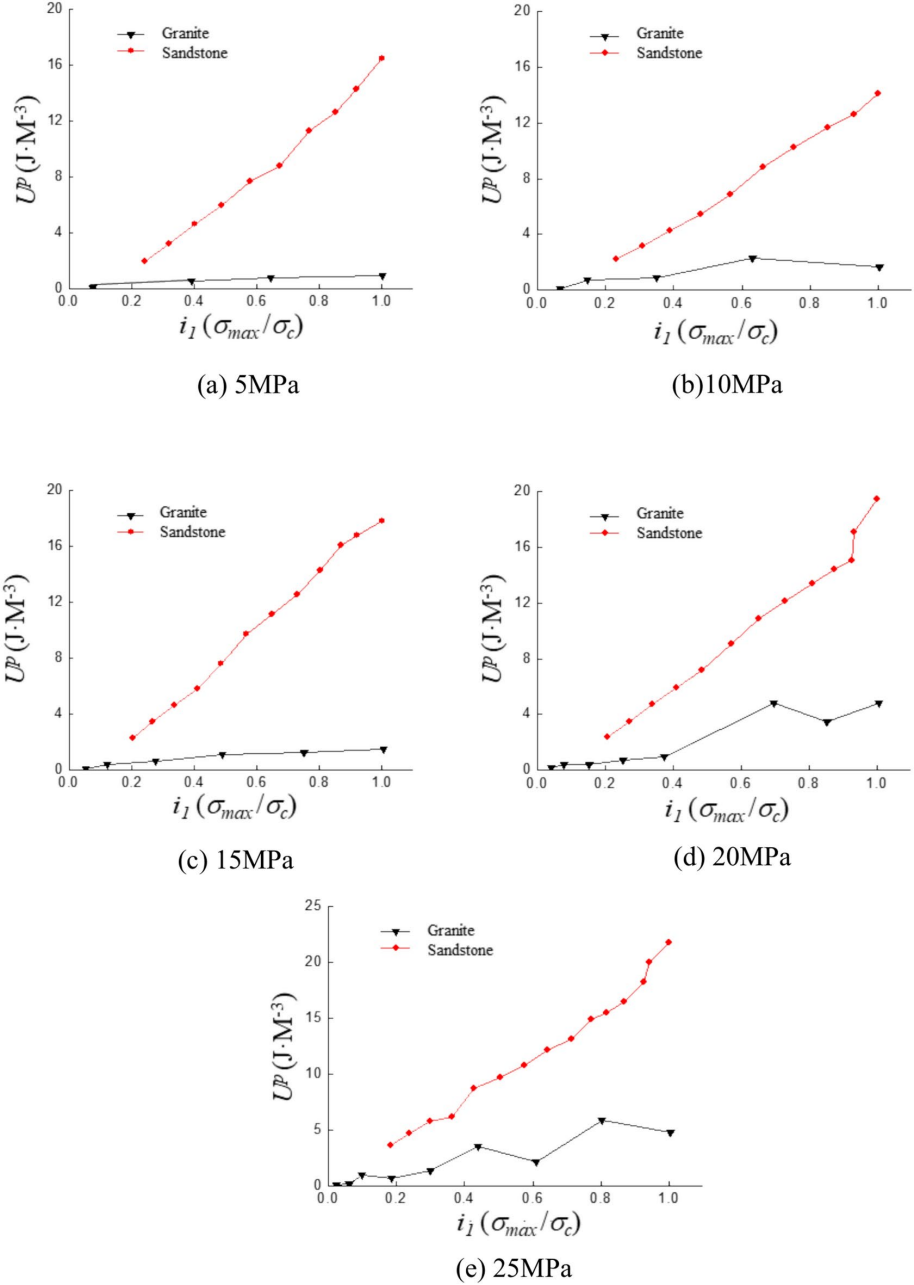
Curve of plastic energy with stress ratio under different confining pressure.

Both sandstone and granite exhibit an increase in plasticity with the rise in stress ratio. The slope of plasticity for sandstone with respect to the stress ratio is 0.70 as shown in Fig. 14a. The slope of granite is 0.09. In Fig. 14b, the slope for sandstone is 0.62 and for granite is 0.17. In Fig. 14c, the slope for sandstone is 0.90 and for granite is 0.18. In Fig. 14d, the slope for sandstone is 0.57 and for granite is 0.17. The increase in plasticity energy for sandstone remains relatively stable with slopes ranging from 0.6 to 0.9. The slopes for granite consistently range between 0.1 and 0.2 in Fig. 14e, with a more gradual increase. The plasticity of sandstone and granite shows a stable growth trend as the increase of stress ratio.

Plastic deformation is an irreversible process, resulting in the generation of plasticity during deformation. As the stress ratio increases in this experiment, internal fractures in the sample gradually form and expand. The increase in internal damage causes the sample to exhibit stronger plasticity. The plastic deformation of the sample occurs more uniformly. The rate of increase in plasticity for sandstone is significantly higher than that of granite. The porosity of sandstone is significantly higher than that of granite. The cementation between the particles of sandstone is weaker than the chemical bonding between the grains of granite.

## Effect of different confining pressures on energy variation

Confining pressure is a fundamental condition that rocks naturally undergo, and most engineering projects involve rocks subjected to confined pressure. The influence of confining pressure aligns more closely with real-world conditions, offering a more accurate reflection of the changes in various parameters of the rock’s actual state. Therefore, this section examines the impact of different confining pressures on the variation of energy parameters and analyzes the underlying reasons for these effects.

### Dissipated energy

Both confining pressure and loading rate will affect the dissipated energy. The crack propagation within the rock occurs more slowly at lower loading rates, resulting in pronounced plastic behavior and greater energy dissipation. Cracks propagate rapidly at higher loading rates, making the rock more susceptible to brittle failure, leading to the rapid accumulation and release of energy with lower dissipation. An increase in confining pressure inhibits crack propagation and enhances energy dissipation. In this test, the loading rate is kept constant at 0.5 mm/min, which has little effect on the dissipated energy and crack extension and is therefore ignored. Therefore, the focus is on studying the variation of dissipated energy with confining pressure.

**Fig. 15:**
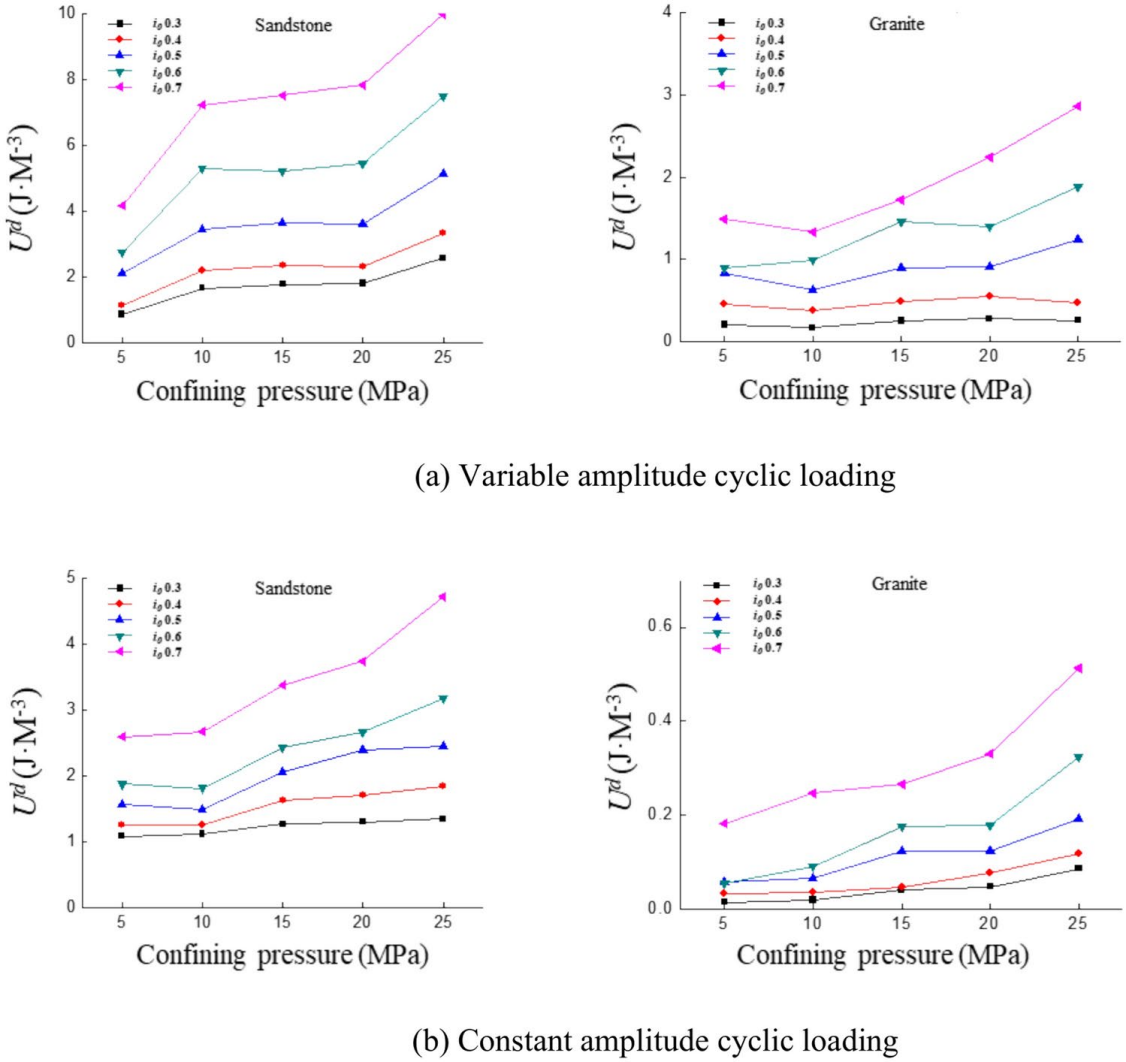
Curve of dissipation energy with confining pressure under different cyclic loading conditions.

As shown in Fig. 15a, it can be observed that when the confining pressure increases from 5 to 10 MPa, the dissipated energy for sandstone increases with the confining pressure. When the confining pressure exceeds 5 MPa, the variation trends of dissipated energy under different stress amplitudes diverge. For an amplitude of 0.5, the dissipated energy remains stable when the confining pressure is between 10 and 15 MPa. Between 20 and 25 MPa, dissipated energy increases significantly again. But the rate of increase is smaller than that observed when the confining pressure was 5 MPa or 10 MPa. When the amplitude is 0.7, dissipated energy slightly decreases as the confining pressure increases from 10 to 15 MPa. But resumes an increasing trend when the confining pressure reaches 20 MPa. Figure 15b shows that, as the confining pressure increases from 5 to 10 MPa, dissipated energy increases. From 10 to 20 MPa, the rate of increase slows down. At 20 MPa, the rate of increase accelerates again, though still slower than at 5 MPa. The trend for granite is similar to that of sandstone. The rate and magnitude of change for granite are smaller.

Under variable amplitude cyclic loading conditions, the dissipated energy of sandstone increases rapidly when the confining pressure is 5–10 MPa, remains stable when it is 10–20 MPa, and increases rapidly again when it is 20–25 MPa. Granite shows a downward trend at 5–10 MPa and rises slowly at 10–25 MPa. The porosity inside the sandstone is high, and there are many initial microcracks. When the confining pressure is 5–10 MPa, it promotes the closure of pores and the expansion of microcracks. This leads to particle rearrangement, friction slip, and local plastic deformation inside the rock. A large amount of energy is released in the form of dissipated energy, causing a rapid rise in dissipated energy. When the confining pressure is 10–20 MPa, most of the pores inside the sandstone are closed and the crack expansion is inhibited. The energy is mainly stored in the form of elastic energy, resulting in a growth of dissipated energy. The internal bearing capacity of the rock increases when the confining pressure increases to 20 MPa, which in turn increases the slip and friction between particles, leading to a significant increase in the dissipated energy again. Granite has a dense and hard structure, low initial porosity and few microcracks. The effect of the confining pressure causes the existing cracks to close when the confining pressure is 5–10 MPa, the energy is stored in the form of elastic energy and the dissipated energy is reduced. When the confining pressure is 10–25 MPa, the crack propagation of granite is inhibited. A small amount of grain fracture, interface slip and local shearing occurs inside the rock as the number of cyclic loading increases, resulting in the release of part of the energy in the form of dissipated energy. Therefore, the dissipated energy shows a slow upward trend but the increase is smaller than that of sandstone.

Each loading and unloading cycle during cyclic loading cause the accumulation and release of energy. Confining pressure provides lateral restraint to the rock, limiting its transverse expansion and requiring greater resistance for crack propagation^54^. Crack propagation is suppressed at higher confining pressures, resulting in fewer new cracks and restricted growth of existing ones during each cycle^55^. Therefore, the energy dissipation due to crack growth under confining pressure will be reduced. Sandstone has more pores and a looser structure, while granite has a denser structure and stronger bonding. The pores of sandstone may be compressed under high confining pressure, resulting in reduced energy dissipation in the initial stage^56^. In contrast, granite’s higher inherent strength may require greater stress to initiate cracking, and the presence of confining pressure further inhibits crack propagation, leading to lower energy dissipation compared to sandstone. Total energy dissipation is primarily associated with internal friction, crack initiation and crack propagation during deformation^57^. Increasing confining pressure promotes a transition from brittle to plastic failure in the rock, resulting in greater energy absorption by irreversible deformation mechanisms such as microcrack slippage and grain rearrangement, rather than energy release through crack propagation^56^. This contributes to an overall increase in total energy dissipation.

The above phenomenon suggests that dissipated energy generally increases with the increase in confining pressure before rock is damaged. However, the rate of increase diminishes as confining pressure rises. This occurs because the confining pressure limits radial deformation and enhances the rock’s load-bearing capacity. It makes internal damage less likely to occur. When the confining pressure is between 5 and 10 MPa, the rate of increase in dissipated energy is faster. This can be understood as the confining pressure’s limitation on radial deformation being weaker. Its impact on the rock’s internal cracks and resistance to deformation is less significant. As the confining pressure increases, this effect becomes more pronounced. The strength of rock increases significantly, and its resistance to deformation also increases. The energy required for crack propagation rises substantially. It results in a greater increase in dissipated energy.

### Elastic energy

**Fig. 16:**
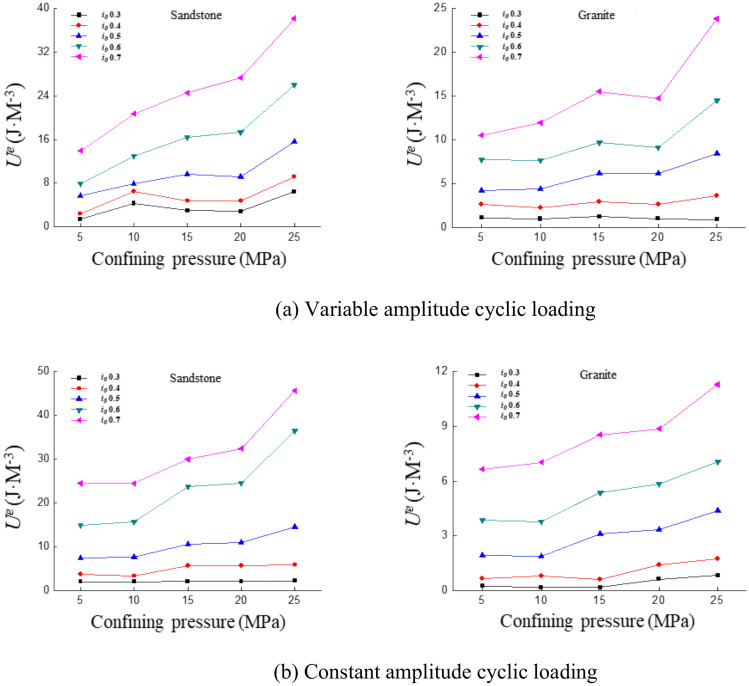
Curve of elastic energy with confining pressure under different cyclic loading conditions.

In section "Dissipated energy", it was observed that dissipated energy increases with confining pressure. As shown in Fig. 16, both sandstone and granite exhibit an increase in elastic energy with rising confining pressure. The pores inside the rock are compressed as the confining pressure increases, resulting in a decrease in its porosity and an increase in its strength. This causes the stress on the rock to increase in the elastic stage, so the elastic energy increases with the increase of confining pressure. Nearly identical patterns were observed under both variable amplitude cyclic loading and constant amplitude cyclic loading. A detailed description is provided for sandstone in Fig. 16a, which represents the changes for both loading conditions and rock types. Between 0 and 20 MPa, the rate of increase in elastic energy for sandstone with confining pressure is gradual. The fitted slope of 0.75. The slope of the elastic energy increase becomes 1.11, when the confining pressure increases from 20 to 25 MPa. The increase in slope is 48%. From the curves in Fig. 16a,b, it can be concluded that the elastic energy increases gradually at a slower rate between 0 and 20 MPa. When the confining pressure rises from 20 to 25 MPa, the rate of increase in elastic energy accelerates.

The elastic energy tends to increase with the increase of confining pressure. When the confining pressure is 5–20 MPa, the elastic energy increases at a lower rate with the confining pressure, while when the confining pressure is 20–25 MPa, the elastic energy increases at a higher rate with the confining pressure. This phenomenon can be attributed to the suppression of internal crack propagation within the rock under a confining pressure of 5–20 MPa, leading to an increase in overall stiffness and a relatively lower rate of elastic energy accumulation. Once the confining pressure reaches 20 MPa, the rock accumulates a significant amount of elastic energy before transitioning into the plastic deformation stage, which is subsequently released upon final failure. Consequently, the increase in elastic energy becomes more pronounced.

As the confining pressure increases, it not only enhances the strength of the sample but also improves its resistance to deformation. The confining pressure input into the system is also converted into energy input into the sample, which increases the degree of particle compaction within the sample. This compaction stores energy within the sample due to the confining pressure, the energy cannot be released. The elastic energy within the sample gradually increases. The rate of increase in elastic energy becomes more pronounced as the confining pressure continues to rise.

### Plasticity energy

**Fig. 17:**
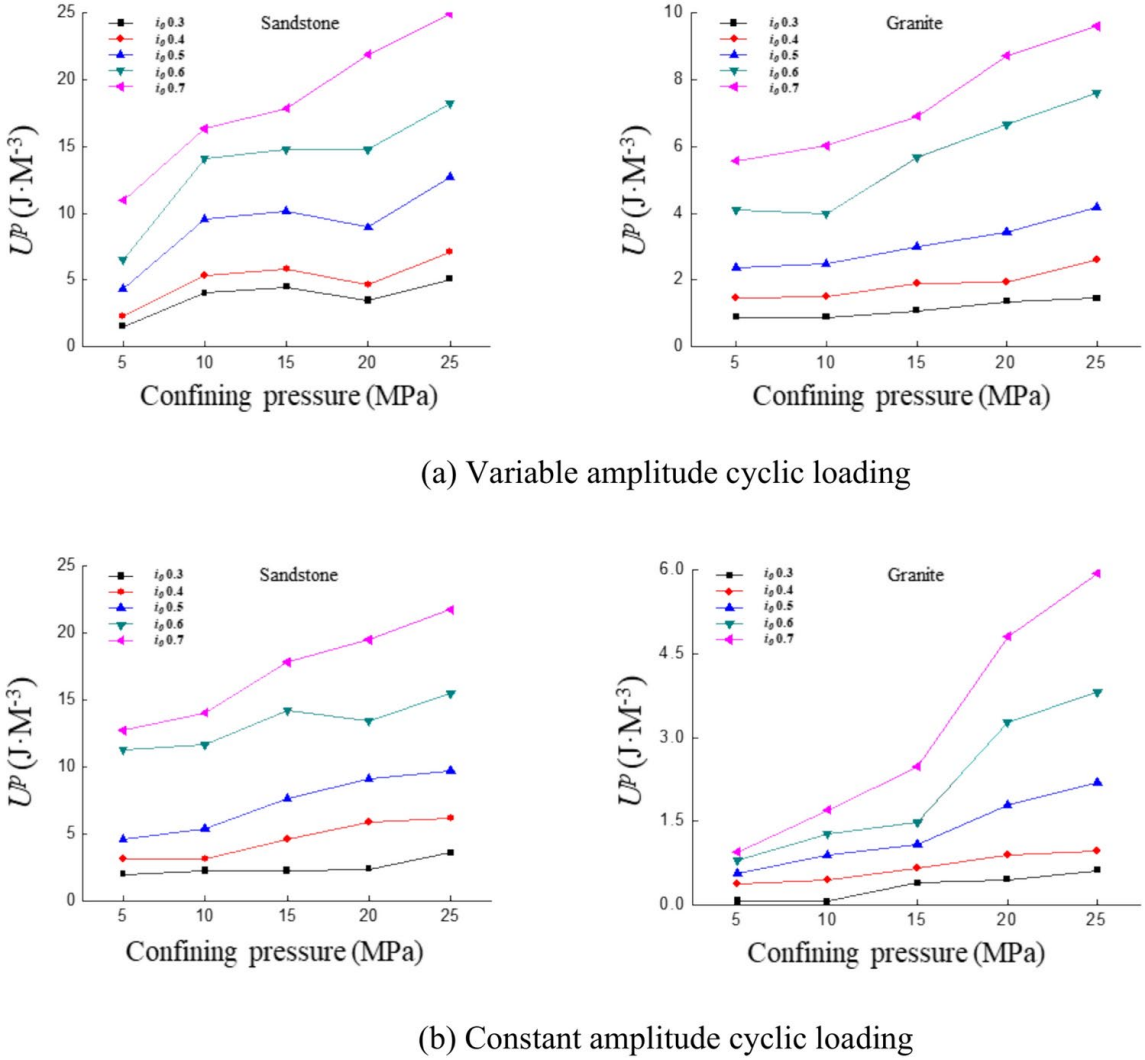
Curve of plastic properties with confining pressure under different cyclic loading conditions.

The plasticity of sandstone exhibits minimal variation with confining pressure at low strain ratios under variable amplitude cyclic loading, as shown in Fig. 17a. The plasticity of sandstone increases more significantly with rising confining pressure as the strain ratio increases. The plasticity variation trend in granite is very similar to that of sandstone, but both the values and the rate of increase are smaller in granite. Under constant amplitude cyclic loading, the increase in plasticity in sandstone remains relatively steady, while the rate of increase in granite accelerates, as shown in Fig. 17b. Although the numerical value of granite’s plasticity remains smaller than that of sandstone, the rate of increase shows a clear upward trend.

Under variable amplitude cyclic loading conditions, the plastic energy of sandstone shows a trend of first rising rapidly, then stabilizing and finally rising rapidly again with confining pressure. The plastic energy of granite tends to be stable with confining pressure. Under constant amplitude cyclic loading conditions, the rate of increase of the plastic energy of sandstone with confining pressure tends to be stable. There is a turning point in the plastic energy of granite with confining pressure, with a small increase rate at 5–15 MPa and a large increase rate at 15–20 MPa. The plastic deformation of sandstone is primarily governed by crack propagation and particle sliding, exhibiting more pronounced nonlinear variations. The higher density and strength of granite result in a more stable response to plastic deformation, with significant changes occurring only under high confining pressure.

The impact of confining pressure on the experiment has been discussed earlier. The sample’s ability to resist deformation is enhanced under the influence of confining pressure. The internal cracks and pores in the sample close more tightly. The energy required to apply the same strain amplitude increases and the portion converted to plasticity also increases. From the deformation point of view, the samples subjected to confining pressure will undergo greater irrecoverable deformation at the same strain amplitude. Rock is compacted and the yield strength increases as the confining pressure increases. The increase of confining pressure limits the crack propagation of rock, resulting in stress concentration at the crack tip inside the rock, which promotes plastic deformation. Therefore, the plastic energy increases with the increase of confining pressure.

## Conclusion

This paper mainly studies the energy changes of sandstone and granite in variable amplitude and constant amplitude cyclic loading tests. The main conclusions are as follows:

(1) A series of variable amplitude stepwise cyclic loading tests were carried out to study the changes of dissipated energy, elastic energy and plastic energy of sandstone and granite with strain ratio, confining pressure and number of cycles. The results show that the dissipated energy, elastic energy and plastic energy of sandstone and granite increase with the increase of strain ratio. The dissipated energy increases with strain amplitude at a higher rate in sandstone than in granite under the same strain ratio. Under the condition of the same strain ratio, the plastic energy of sandstone is generally greater than the elastic energy of granite.

(2) A series of constant amplitude stepwise cyclic loading tests were carried out to study the energy change law of sandstone and granite in constant amplitude stepwise cyclic loading tests. The results show that with the increase of stress ratio, the dissipated energy, elastic energy and plastic energy of sandstone and granite gradually increase. The dissipation energy, elastic energy, and plastic energy of sandstone increase at a higher rate than those of granite as the stress ratio rises. When the stress ratio ranges from 0.85 to 0.95, a sudden change in dissipated energy occurs, with the rate of energy increase being 6 to 12 times greater than before. The increase rate of plastic energy for sandstone ranges from 0.6 to 0.9, while for granite, it ranges from 0.1 to 0.2.

(3) In the graded cyclic loading tests under different confining pressures, the dissipated energy, elastic energy, and plastic properties of sandstone and granite all tend to increase with the increase of confining pressure. The confining pressure limits the axial deformation of the specimen. The dissipation energy, elastic energy and plastic energy of sandstone exhibit a pronounced increasing trend with rising confining pressure and strain ratio. In contrast, the values and growth rates of dissipation energy, elastic energy and plastic energy in granite remain relatively small under the same conditions.

## Data Availability

The datasets used and/or analysed during the current study available from the corresponding author on reasonable request.
